# Morphologic mimicry in high-grade lung carcinoma: a case report of RB1-intact, MYC-amplified, and NFE2L2-mutated pseudo-small cell lung cancer

**DOI:** 10.3389/fonc.2026.1807289

**Published:** 2026-05-19

**Authors:** Furong Wang, Yaxian Yang, Zhijian Luo, Weiquan Luo, Jian Huang

**Affiliations:** 1 Department of Pathology, Huizhou Central People's Hospital, Huizhou, China; 2 Pathology Discipline Development Center, Guangzhou Huayin Health Medical Group Co., Ltd, Guangzhou, China; 3 Department of Medical Oncology, Huizhou Central People's Hospital, Huizhou, China; 4 Subspecialty Laboratory, Guangzhou Huayin Health Medical Group Co., Ltd, Guangzhou, China; 5 Department of Pathology, Affiliated Hospital of Guangdong Medical University, Zhanjiang, China

**Keywords:** lineage plasticity, *MYC* amplification, *NFE2L2*, precision medicine, *RB1*, small cell lung cancer

## Abstract

Small cell lung cancer (SCLC) is historically defined by distinct neuroendocrine morphology and the obligate bi-allelic inactivation of *TP53* and *RB1*. However, the advent of comprehensive molecular profiling has revealed a subset of high-grade tumors that morphologically mimic SCLC but lack its canonical genomic signatures. These “pseudo-SCLC” cases present significant diagnostic and therapeutic challenges. A 58-year-old male presented with a scapular lytic lesion and pulmonary opacities. Histopathology of the bone biopsy revealed small round blue cells with nuclear molding, crush artifact, and a Ki-67 index of 90%, strongly suggestive of SCLC. Paradoxically, immunohistochemistry (IHC) demonstrated a “null” neuroendocrine phenotype (CD56-, CgA-, Syn focal+) while retaining TTF-1 expression. Next-Generation Sequencing (NGS) identified a pathogenic *TP53* mutation (p.V173L) and high-level *MYC* amplification (CNV: 5×). Crucially, the tumor retained wild-type *RB1*. Furthermore, a pathogenic *NFE2L2* mutation (p.D29V)—a genetic hallmark typically associated with oxidative stress response in non-small cell lung cancer (NSCLC) was identified. This case suggests a potential association with *MYC*-driven lineage plasticity, where a solid-type adenocarcinoma may undergo de-differentiation into an SCLC-like phenotype. The retention of *RB1* and the presence of the *NFE2L2* mutation distinguish this entity from classic SCLC, supporting a reclassification as high-grade NSCLC. Recognition of this molecular subset is vital, as *NFE2L2* mutations are theoretically linked to resistance to standard platinum-based regimens, potentially necessitating therapeutic strategies distinct from standard SCLC algorithms.

## 1 Introduction

Small cell lung cancer (SCLC) is an aggressive malignancy traditionally diagnosed by its characteristic morphology and the expression of neuroendocrine (NE) markers. Its morphological features include small cells with scant cytoplasm, nuclear molding, and “salt-and-pepper” chromatin (1). Genomically, classic SCLC is uniformly defined by the universal inactivation of *TP53* and *RB1* (>95% of cases) (2).

However, the binary classification of lung cancer is increasingly challenged by tumors residing in the “grey zone.” Recent genomic studies have identified subtypes of high-grade neuroendocrine carcinomas that are NE-low and, crucially, retain functional RB1 protein (3). Additionally, *MYC* family gene amplification has been implicated in driving lineage plasticity, potentially allowing non-small cell lung cancer (NSCLC) cells to acquire SCLC-like morphology through de-differentiation (4).

We report a diagnostic challenge involving a high-grade metastatic carcinoma Although its morphology resembled SCLC, its immunophenotype and genomic profile contradicted this diagnosis. We highlight the critical role of integrating *RB1* status and *NFE2L2* mutations to correctly classify these “pseudo-SCLC” cases and guide appropriate therapy.

## 2 Case description

**Figure 1:**
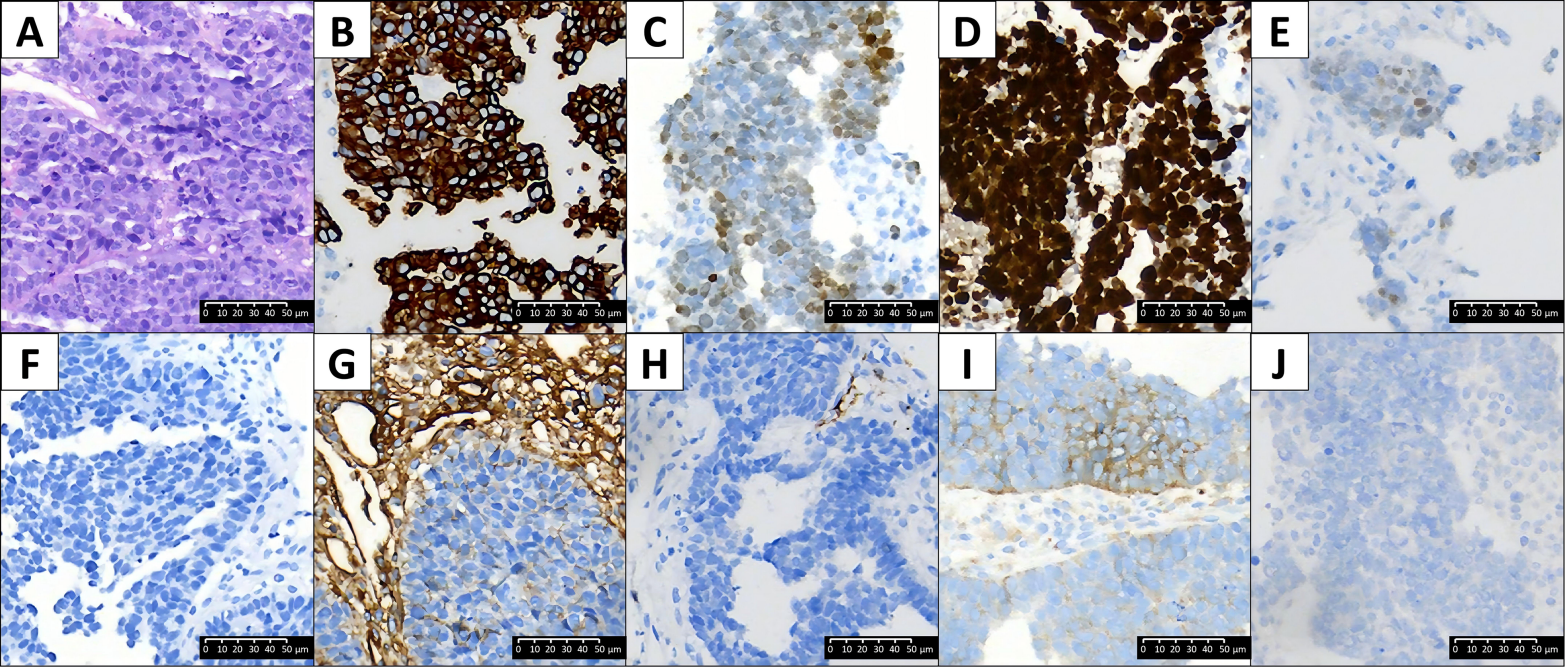
Histological and immunohistochemical features **(A)** H&E staining (400×) reveals sheets of cells with a high nuclear-to-cytoplasmic ratio, nuclear molding, and “salt-and-pepper” chromatin, morphologically mimicking SCLC. **(B–J)** (400×) Immunohistochemistry montage: The tumor cells are positive for **(B)** (400×)CK(pan) and **(C)** (400×)TTF-1, **(D)** (400×) diffuse high Ki-67 expression (>90%), **(E, F)** (400×)but negative for CD56 and Chromogranin A (CgA). **(G)** (400×) Synaptophysin (Syn) showed focal positivity, **(H)** (400×) INSM1 was shown weakly positive, and **(I)** (400×) CD99 shows heterogeneous, membranous positivity in the tumor cells. The tumor was negative for **(J)**P40, CK5/6, and LCA (not shown).

A 58-year-old non-smoking male presented with progressive pain in the left scapula. Computed Tomography (CT) revealed lytic destruction of the left scapula and multiple patchy shadows in bilateral lungs. A CT-guided biopsy of the scapular lesion was performed. Hematoxylin and Eosin (H&E) staining showed sheets and nests of small-to-medium-sized tumor cells with scant cytoplasm and hyperchromatic nuclei(as shown in **Figure 1A**). Prominent nuclear molding and extensive crush artifacts were observed (**Figure 1A**). Mitotic figures were abundant. The morphological impression was strongly suggestive of Small Cell Lung Cancer. IHC profiling revealed a discordant pattern. Tumor cells were positive for CK (pan) (**Figure 1B**). and TTF-1 (**Figure 1C**), confirming a lung origin. The Ki-67 proliferation index was approximately 90% (**Figure 1D**), consistent with high-grade carcinoma. However, classic neuroendocrine markers CD56 and Chromogranin A (CgA) were negative (**Figures 1E, F**). Synaptophysin (Syn) showed only focal positivity (**Figure 1G**), and INSM1 was weakly positive (**Figure 1H**). Additionally, CD99 showed heterogeneous membranous positivity (**Figure 1I**). The tumor was negative for P40 (**Figure 1J**), CK5/6, and LCA (data not shown).

Given the discordance between the SCLC-like morphology and the NE-low immunophenotype, hybrid capture-based Next-Generation Sequencing (NGS) covering 1021 cancer-related genes was performed (**Table 1** summarizes the genomic alterations). The analysis revealed a driver mutation profile characterized by pathogenic alterations in *TP53* (p.V173L; VAF 54.7%) and *NFE2L2* (p.D29V; VAF 71.5%). A focal *MYC* amplification (copy number 5) was identified, alongside additional sequence variants in *CTNNB1* (p.S37F) and *PTPN11* (p.A72S). Notably, *RB1* was confirmed to be wild-type and intact. No actionable alterations were detected in *EGFR*, *ALK*, *ROS1*, or *KRAS*. The tumor was microsatellite stable (MSS) and exhibited a low tumor mutational burden (TMB) of 6.15 Muts/Mb.

**Table 1:** The mutation of genes detected in FFPE tissue.

GENE	FEATUREID	EXON	HGVS_C	HGVS_P	TYPE	AF	Clinical level
*TP53*	NM_000546.6	exon5	c.517G>T	p.V173L	missense	54.67%	Class II variants
*CTNNB1*	NM_001904.4	exon3	c.110C>T	p.S37F	missense	50.72%	
*NFE2L2*	NM_006164.	exon2	c.86A>T	p.D29V	missense	71.5%	
*MYC*	/	/	/	/	amplification	5×	
*PTPN11*	NM_002834.5	exon3	c.214G>T	p.A72S	missense	53.45%	
CTNNB1	NM_001904.4	exon13	c.1955-13_1959del	/	Splicing	19.91%	Class III variants
FH	NM_000143.4	exon8	c.1136C>T	p.A379V	missense	21.11%	
NFE2L2	NM_006164.5	exon5	c.1495C>T	p.R499W	missense	70.59%	
SLX4	NM_032444.4	exon12	c.4114C>G	p.R1372G	missense	26.86%	
SMAD4	NM_005359.6	exon10	c.1194G>C	p.W398C	missense	15.06%	
CTNNB1	NM_001904.4	exon13	c.1955-13_1959del	/	Splicing	19.91%	

HGVS_C, Human Genome Variation Society Coding DNA (sequence) Nomenclature; HGVS_P, Human Genome Variation Society Protein (sequence) Nomenclature; AF, Allele Frequency.

Based on the integrated genomic (*RB1*-intact, *NFE2L2*-mutated) and pathologic data, the diagnosis was revised to High-Grade Lung Carcinoma with Neuroendocrine Differentiation (Non-SCLC type), favoring a solid adenocarcinoma origin undergoing high-grade transformation.

## 3 Discussion

This case exemplifies the potential pitfalls of relying solely on morphology for the diagnosis of high-grade thoracic malignancies. While the H&E appearance mimicked SCLC, the genomic fingerprint—specifically the retention of *RB1*, *MYC* amplification, and *NFE2L2* mutation—suggested a biological identity distinct from classic SCLC.

The inactivation of *RB1* is the hallmark of SCLC. Seminal work by Rekhtman et al. proposed that *RB1*-retained high-grade neuroendocrine carcinomas are biologically distinct from SCLC and genetically closer to NSCLC (specifically Large Cell Neuroendocrine Carcinoma, LCNEC, or solid adenocarcinoma) (5). They identified two distinct genomic subsets of Large Cell Neuroendocrine Carcinoma (LCNEC): an “SCLC-like”type characterized by concurrent TP53 and RB1 loss, and an “NSCLC-like” type (mainly resembling adenocarcinoma) characterized by the retention of functional RB1 and mutations in genes such as STK11, KRAS, or NFE2L2 (3, 5). Our case fits the “NSCLC-like” molecular profile due to the wild-type RB1 status and the presence of a truncal *NFE2L2* mutation. This molecular framework leads us to propose the hypothesis that despite the “pseudo-SCLC” morphology, the tumor might represent a highly dedifferentiated NSCLC lineage that has acquired neuroendocrine features through lineage plasticity. While plausible, this interpretation remains exploratory and distinct from the definitive genomic diagnosis of *de novo* SCLC.

*MYC* amplification has been identified as a significant factor in tumor aggression and plasticity (6). In mouse models, *Myc* overexpression combined with *Tp53* loss promotes a variant of SCLC that is typically “Neuroendocrine-Low” (low expression of ASCL1/NEUROD1) (7). This aligns with our patient’s immunoprofile: a Ki-67 index of 90% (driven by *MYC*) but negativity for CD56 and CgA. The *MYC* amplification is hypothesized to have contributed to the de-differentiation of the original adenocarcinoma cells into a stem-like state, manifesting as small cells with CD99 expression (a marker of primitive neuroectodermal differentiation). In the absence of functional validation, this remains a speculative mechanism to explain the observed “morphologic mimicry.”

A clinically pivotal finding in this case is the *NFE2L2* p.D29V mutation. *NFE2L2* (encoding NRF2) mutations are found in ~15-20% of lung squamous cell carcinomas and solid-type adenocarcinomas but are extremely rare in *de novo* SCLC (<5%) (8). The identification of *NFE2L2* p.D29V at a high variant allele frequency (71.5%) suggests it is a truncal event, pointing towards an NSCLC origin (9). Clinically, constitutive activation of the NRF2 pathway upregulates antioxidant enzymes, protecting tumor cells from oxidative stress induced by cytotoxic chemotherapy (especially platinum agents) and radiotherapy. Consequently, this mutation is theoretically suggestive of a poor response to the standard etoposide-platinum (EP) regimen used for SCLC. However, it is important to distinguish this hypothesis-generating interpretation from clinical evidence, as the actual predictive value of NFE2L2 in this rare subset requires further investigation.

Correct classification of this tumor as an *RB1*-intact, *NFE2L2*-mutated high-grade carcinoma is crucial. Patients with *RB1*-wild-type LCNEC or high-grade carcinomas have been shown to respond better to NSCLC-like regimens (e.g., Gemcitabine/Taxane + Platinum) than to SCLC regimens (EP) (10). Furthermore, considering the potential *NFE2L2*-mediated resistance mechanism, close monitoring for early progression is warranted until more robust clinical evidence becomes available.

In conclusion, this case report provides a descriptive account of a high-grade lung carcinoma that morphologically mimics SCLC but possesses an “NSCLC-like” molecular signature (*RB1*-intact, *NFE2L2-*mutated). While our findings suggest a possible role for MYC-driven lineage plasticity, these interpretations remain exploratory. This case highlights the necessity of integrated diagnostic approaches; however, prospective studies are essential to validate these molecular markers as definitive therapeutic guides.

## Data Availability

The original contributions presented in the study are included in the article/supplementary material. Further inquiries can be directed to the corresponding author.
